# Structural Collapse of Mental Health Service Provision in Gaza: A Service Evaluation Under Active Conflict

**DOI:** 10.1192/bjo.2026.11525

**Published:** 2026-06-30

**Authors:** Kholoud Abuhjayyer, Khalil Ibrahim Hamad, Rawia Hamam, Mohammed Abdulrahman, Abdullah Aljamal

**Affiliations:** 1 Applied Child Psychology (MSc) Student, Brighton, United Kingdom; 2 UNRWA-GFO, Gaza, Palestine; 3 Former Co-Chair, MHPSS Technical Working Group (2025), Gaza, Palestine; 4 Gaza Community Mental Health Programme, Gaza, Palestine; 5 Co-Chair, MHPSS TWG, Gaza, Palestine; 6 Mental Health Consultant, Gaza, Palestine; 7 General Administrator of Mental Health Services–Ministry of Health, Gaza, Palestine

## Abstract

**Aims:**

To systematically evaluate the structural and functional capacity of mental health services in Gaza following over 2 years of active conflict (Oct 2023–Jan 2026), quantify infrastructure and workforce losses, and identify critical service gaps requiring humanitarian response prioritization.

**Methods:**

This service evaluation utilized triangulated data sources: (a) official Ministry of Health Mental Health Department records (Oct 2023–Jan 2026); (b) field observations carried out by World Health Organization (WHO) fieldworkers (Oct 2024–Nov 2025); (c) Mental Health and Psychosocial Support (MHPSS) Technical Working Group coordination data and facility assessments (Oct 2023–Jan 2026). The evaluation was conducted using standardised MHPSS assessment toolkits adapted to the local context. Evaluation scope encompassed all governmental psychiatric facilities, community mental health centres, selected local and international non-governmental organisation (NGO) service points, workforce capacity, medication supply infrastructure, and service accessibility across Gaza Strip. Analysis employed descriptive statistics and thematic categorization of operational barriers. Data sources comprised aggregated, non-identifiable service statistics and infrastructure assessments.

**Results:**

Gaza's sole functional psychiatric hospital (39-bed capacity, 2,200 annual emergency presentations, 719 annual admissions pre-conflict) sustained structural damage, rendering it non-operational since November 2023. A replacement psychiatric hospital that was planned before the war and was due to open in 2024 sustained damage before activation. All six governmental Community Mental Health Centres (serving approximately 100,000 annual service users) were destroyed (during April 2024–2025).

Workforce capacity declined critically: over 10 mental health professionals were confirmed killed, with only three Board-certified practicing psychiatrists remaining in Gaza (two more projected upon completion of the current training cohort in 2026). Pre-war, there was a single Board-certified child and adolescent psychiatrist, currently unable to practice within Gaza. Multiple NGO facilities sustained destruction or damage. Psychotropic medication supply chains were severely disrupted following the destruction of the WHO's pharmaceutical warehouse. Health information infrastructure, including electronic medical record systems and referral pathways, became non-functional. Several key service restoration priorities were identified, including: establishing minimum psychiatric referral capacity for acute presentations; securing sustainable psychotropic medication supply and implementing clinical supervision and psychological support systems for the remaining workforce.

**Conclusion:**

Findings demonstrate structural collapse of specialized mental health service capacity. Feasible mechanisms to support the identified priorities for service restoration include integrating MH inpatient services in primary and secondary health facilities, context-specific capacity building, international tele-supervision partnerships and workforce protection measures as integrated response components.

